# Investigating spontaneous retinal venous pulsation using Doppler optical coherence tomography

**DOI:** 10.1038/s41598-019-40961-4

**Published:** 2019-03-12

**Authors:** Andreas Wartak, Florian Beer, Sylvia Desissaire, Bernhard Baumann, Michael Pircher, Christoph K. Hitzenberger

**Affiliations:** Center for Medical Physics and Biomedical Engineering, Medical University of Vienna, Vienna, 1090 Austria

## Abstract

We demonstrate the advantages of optical coherence tomography (OCT) imaging for investigation of spontaneous retinal venous pulsation (SRVP). The pulsatile changes in venous vessel caliber are analyzed qualitatively and quantitatively using conventional intensity-based OCT as well as the functional extension Doppler OCT (DOCT). Single-channel and double-channel line scanning protocols of our multi-channel OCT prototype are employed to investigate venous pulsatile caliber oscillations as well as venous flow pulsatility in the eyes of healthy volunteers. A comparison to recordings of scanning laser ophthalmoscopy (SLO) – a standard en-face imaging modality for evaluation of SRVP – is provided, emphasizing the advantages of tomographic image acquisition. To the best of our knowledge, this is the first quantitative time-resolved investigation of SRVP and associated retinal perfusion characteristics using OCT.

## Introduction

Spontaneous retinal venous pulsation (SRVP) describes the phenomenon of rhythmic caliber oscillations of one or multiple major retinal veins at the site of the optic nerve head (ONH)^1,2^. Before forming a single venous trunk just superior to the lamina cribrosa, the inferiorly descending retinal hemiveins are reported to present pulsatile luminal changes in up to 98% of the eyes of healthy human subjects^3–6^. The characteristics of SRVP are claimed to be determined by the transmural pressure (the pressure gradient between internal and external venous pressure) and venous compliance (i.e. elastic properties of vessel wall, vessel diameter, surrounding anatomical embedding/overlying tissue thickness)^1^.

In a simplistic scenario^7^, the intraocular pressure (IOP) is commonly equated with the external venous pressure, while the internal venous pressure is equated with the intracranial pressure (ICP)^1,8^. Thus, reports suggest that the ratio between IOP and ICP may be reflected in the characteristics of SRVP^2,9,10^. More specific, the absence of SRVP is reported to be linked to an increase of ICP at constant IOP^11–14^. Hence, those reports emphasize that a better understanding of SRVP and its detection and characterization in the individual eye, in combination with standard-of-care IOP determination may enable non-invasive ICP assessment – one of the holy grails of neurology. As of today, methods for non-invasive ICP assessment are rare and error-prone^7,15,16^, thus, a more substantial and reliable approach would have immediate impact.

However, SRVP shows strong inter-subject variability, in particular, due to the uniqueness of retinal vasculature features that govern venous compliance. Furthermore, SRVP seems very specific to any hemivenous section itself^1,17^. Thus, the significance of SRVP for potential ICP assessment is questionable and requires a more thorough analysis.

Previous studies of SRVP observed pulsatile venous caliber oscillations either directly, via examination using a conventional ophthalmoscope or a slit lamp^2,5^, or via optical imaging modalities, such as fundus photography/videography^18^ or scanning laser ophthalmoscopy (SLO)^19,20^. These modalities are en-face plane imaging techniques, only providing 2D transverse projection images of the optic disc surface without depth information. However, as suggested by previous reports and demonstrated by mathematical simulations, the largest venous caliber change is expected to occur in axial and not transversal direction^21–23^. Only modified photo-plethysmography/densitometry tries to assess variations in the axial blood column over time by monitoring changes of retinal back reflection^24,25^.

However, there exists another highly successful optical imaging modality that enables high speed, high resolution, and, in particular, cross-sectional image acquisition of transparent or translucent samples such as the human retina – optical coherence tomography (OCT)^26^. OCT has revolutionized retinal diagnostics^27^ but until today only been used indirectly or with limited potential to study SRVP. First, the retinal nerve fiber layer thickness, determined by OCT, was reported to correlate with SRVP amplitude^28^. Second, Lam *et al*. investigated the structural characteristics of ONH tissue at the sites of SRVP using a commercially available OCT instrument^17^. However, the important aspect of time-resolved investigations to display SRVP at different cardiac phases was not pursued or mentioned in this article.

In this paper we present, to the best of our knowledge, the first quantitative time-resolved OCT investigations of the phenomenon of SRVP in the eyes of healthy human volunteers. In addition to purely intensity-based imaging, we exploit OCT’s functional extension Doppler OCT (DOCT)^29^ to increase detection sensitivity of SRVP. DOCT’s additional functional image contrast enables qualitative as well as quantitative investigations of the retinal sites of venous collapse and the closely surrounding areas in previously unmatched detail.

## Methods

**Figure 1:**
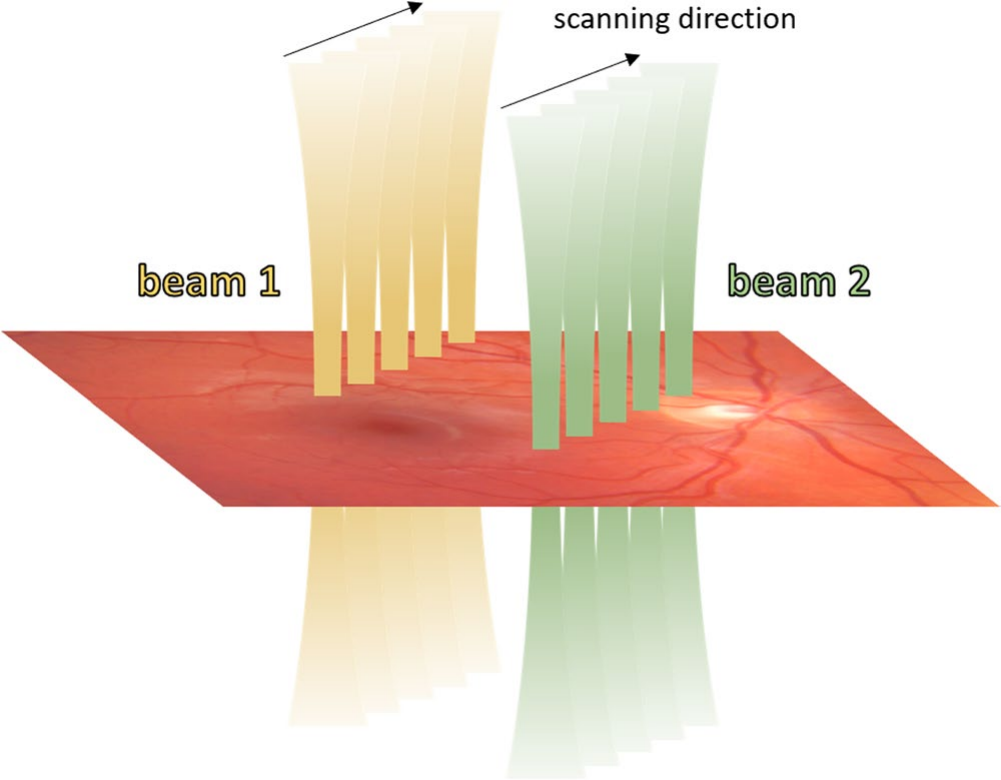
Schematics of parallel line scanning geometry of the double-channel imaging mode used in this work.

All investigations were performed using a custom-built time-encoded multi-channel DOCT prototype described in greater detail elsewhere^30,31^. In short, the system features a tunable light source (central wavelength: *λ*_0_ = 1045 nm; bandwidth: Δ*λ* = 100 nm; sweep/A-scan rate: *f* = 100 kHz) and allows for switching of interferometer sample arms to enable sample illumination by up to three different channels within the same acquisition. However, in this study the system was only operated in single-channel and double-channel mode. Due to the applied switching scheme of inter-A-scan switching^31^, the double-channel mode only permitted an A-scan rate of 50 kHz per channel while for the single-channel mode the full 100 kHz were accessible. In contrast to previous multi-channel operation^30–33^, the individual channels were not probing a mutual sample spot but focused on separate sample locations to enable parallel line scanning^34^ – cf. Fig. 1. The lateral displacement distance of B-scans generated by the individual channels used for this study was 0.9 mm.

The presented study is in agreement with the tenets of the Declaration of Helsinki and was approved by the institutional ethics committee. Prior to the *in vivo* investigations, informed consent was obtained from the healthy human volunteers. The laser safety limits in terms of maximum permissible exposures were met by a maximum beam power of 1.9 mW at the cornea.

Multiple consecutive linear B-scans were recorded at the same retinal location to enable visualization of changes over the cardiac cycle. Different B-scan lengths (0.8, 1.2 mm), sampling densities (1024, 2048 A-scans) and rotational orientations of the scanning line/lines were used throughout the study. Each data recording took between three and five seconds (depending on the exact measurement parameters). Including subject alignment, a single measurement session (consecutive acquisition of multiple data sets) did not exceed ten minutes. The OCT data processing pipeline to generate intensity as well as phase-difference (Doppler) tomograms from raw data, was similar to previously published work^31–33,35^.

Within this pilot study, we show exemplary image data of veins exhibiting SRVP of two eyes of two healthy human volunteers (1 female, age: 29 yrs; 1 male, age: 28 yrs), that were imaged with our multi-channel OCT prototype. For comparison with previous SRVP reports, image data of standard ophthalmic imaging procedures (fundus photography and SLO) of the same eyes were recorded at the Department of Ophthalmology of the Medical University of Vienna. SLO movies were acquired using a commercial device (Spectralis HRA + OCT, Heidelberg Engineering GmbH) using a 15° × 15° scan field at 768 pixels squared and a frame rate of 10 frames per second (fps).

## Results

### Quantification of SRVP

The presented image data of the two eyes showing SRVP illustrate the advantage of cross-sectional image acquisition for investigation of SRVP. Data of the same eyes acquired with multiple ophthalmologic imaging modalities is shown. The OCT data sets in this sub-section were acquired at 100 kHz using the single-channel mode of our instrument.

**Figure 2:**
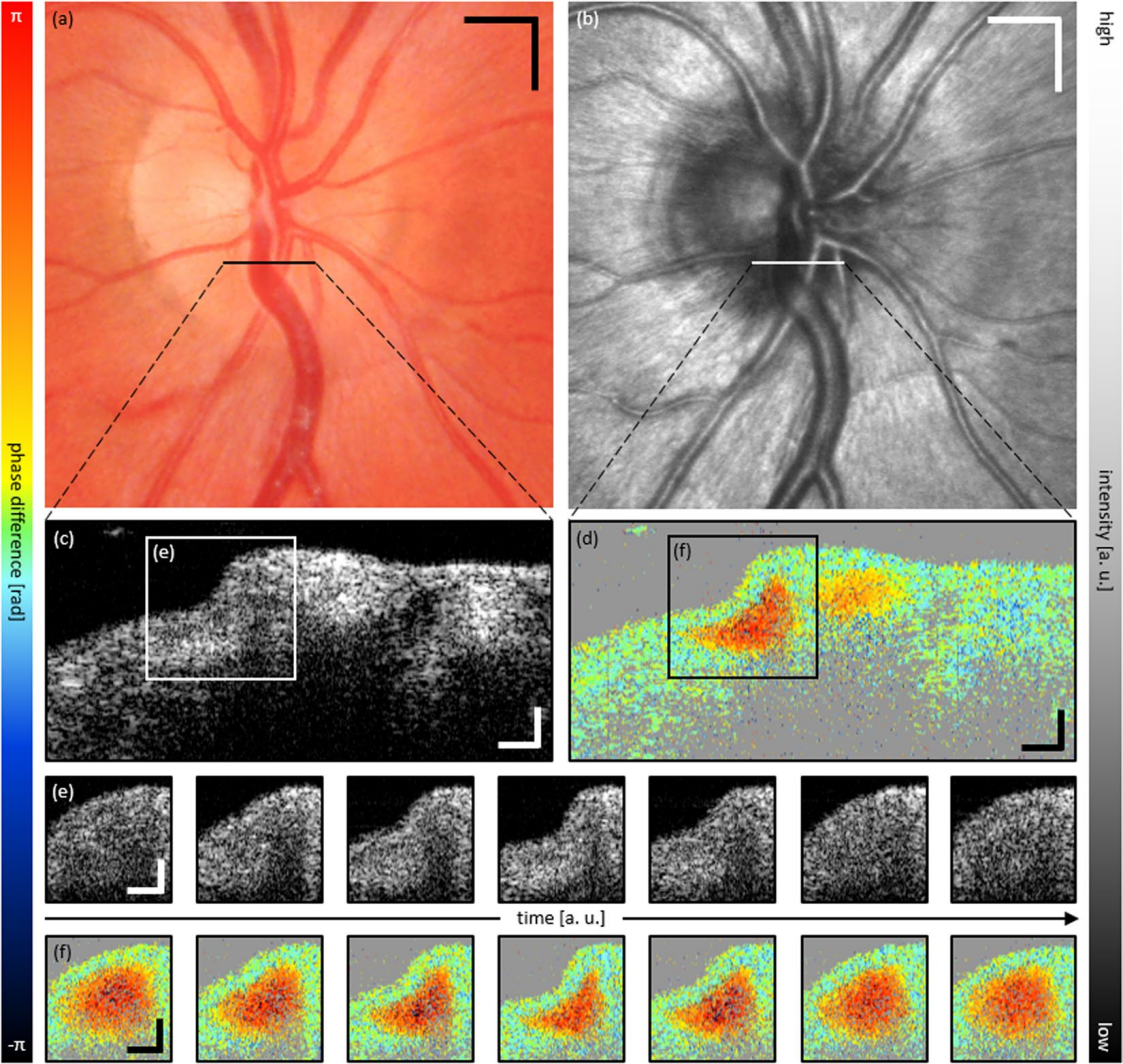
Representative multi-modal image data of SRVP of subject 1. (**a**) Fundus photography and (**b**) SLO image with indicated region of collapse. Cross-sectional OCT (**c**) intensity and (**d**) Doppler tomogram at indicated region of collapse. (**e**,**f**) respective time sequence over full cardiac cycle of ROI indicated in (**c**,**d**). Supplementary Videos S1 and S2 provide movie-files of the snapshot images of (**b**–**d**), respectively. Scale bars: (**a**,**b**): 0.5 mm; (**c**–**f**): 0.1 mm.

Figure 2 depicts en-face images of the ONH region using fundus photography (Fig. 2(a)) and SLO (Fig. 2(b)), respectively. Figure 2(c,d) depict corresponding cross-sectional intensity and Doppler tomograms of the location indicated in Fig. 2(a,b). In this case, the inferior hemivein was observed to be partially collapsing just superior to its crossing with a major retinal artery – at the location of the black/white horizontal line in Fig. 2(a,b). Figure 2(c,d) display tomograms of a cross-section at the site of highest amplitude of collapse. Even though the partial collapse is noticeable in Fig. 2(c,d), only time-resolved image analysis enables clear visualization. Therefore, Fig. 2(e,f) additionally provide a time sequence over a full cardiac cycle of a region of interest (ROI) indicated in Fig. 2(c,d). Although the partial venous collapse can be identified clearly in the intensity tomogram sequence (Fig. 2(e)), only the Doppler tomogram sequence enables a clear differentiation of static retinal tissue from dynamic blood components (Fig. 2(f)). Supplementary Video S1 presents a movie of consecutively acquired tomograms of a full cardiac cycle at the location of the snapshot images of Fig. 2(c,d) including both intensity (top) and Doppler (bottom) image data (44 fps). An SLO movie of another full cardiac cycle of the snapshot image of Fig. 2(b) is presented in Supplementary Video S2 (10 fps).

**Figure 3:**
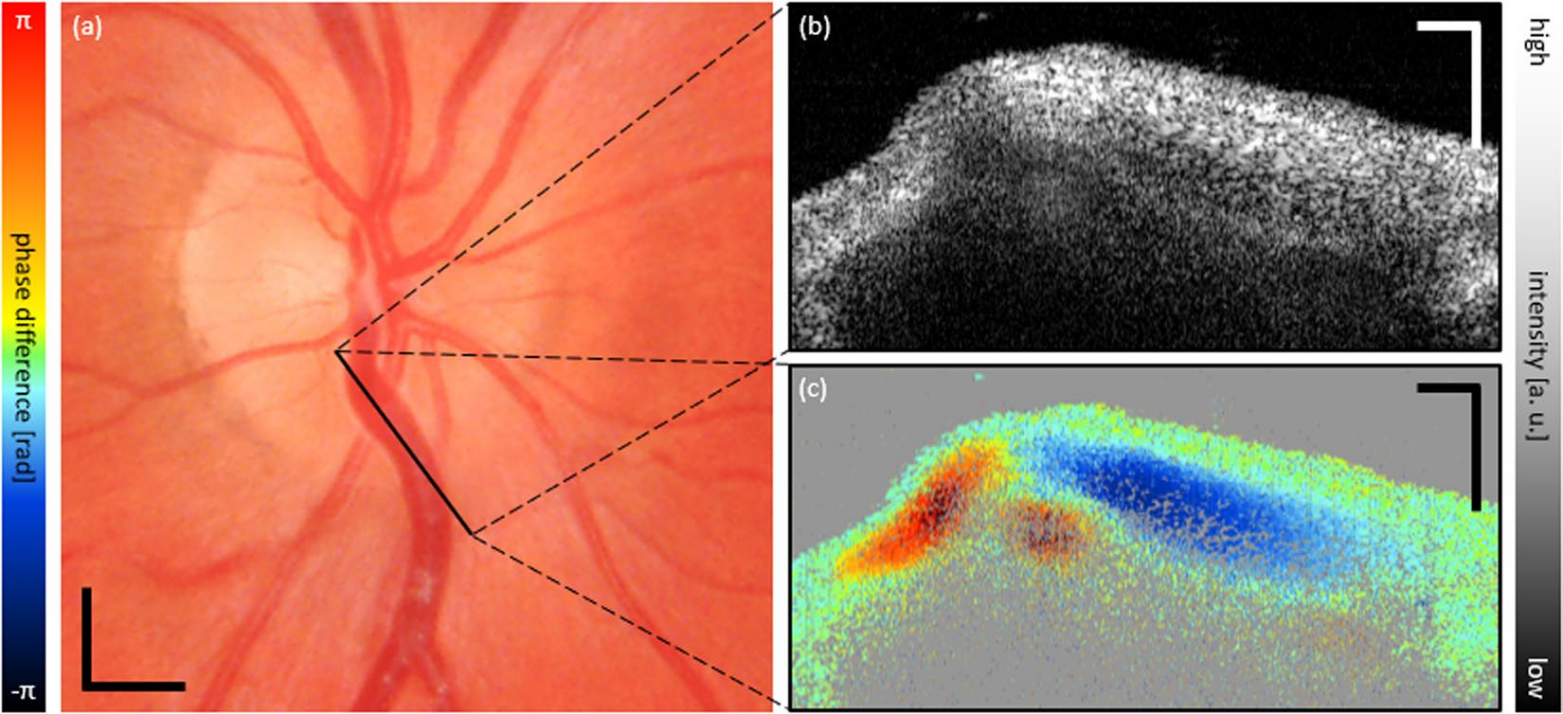
Linear scan along collapsing venous segment. (**a**) Fundus photography with indication of scanned ROI. (**b**) Snapshot of OCT intensity tomogram. (**c**) Corresponding DOCT tomogram showing the venous collapse on the left-hand-side of the image. Supplementary Video S3 provides a movie-file over a full cardiac cycle of the snapshot images of (**b**,**c**). Scale bars: (**a**): 0.5 mm; (**b**,**c**): 0.2 mm.

In addition, we recorded a linear B-scan along the same partially collapsing hemivein – cf. Fig. 3 – and provide a movie of consecutively acquired tomograms of a full cardiac cycle of the snapshot images of Fig. 3(b,c) – cf. Supplementary Video S3. The movie features an intensity (top) as well as a Doppler tomogram (bottom) sequence acquired at 100 kHz A-scan rate using the single-channel mode (24 fps). The location of the partial collapse on the left-hand side of the scan is noticeable on both tomogram types. Since the sign of the inclination of the vein is changing with respect to the orientation of the imaging beam, the Doppler signal changes from shades of orange to shades of blue from left to right with zero phase-difference signal being detected just at the apex on top of the retinal artery running beneath (at this location, the Doppler angle is ~90°).

**Figure 4:**
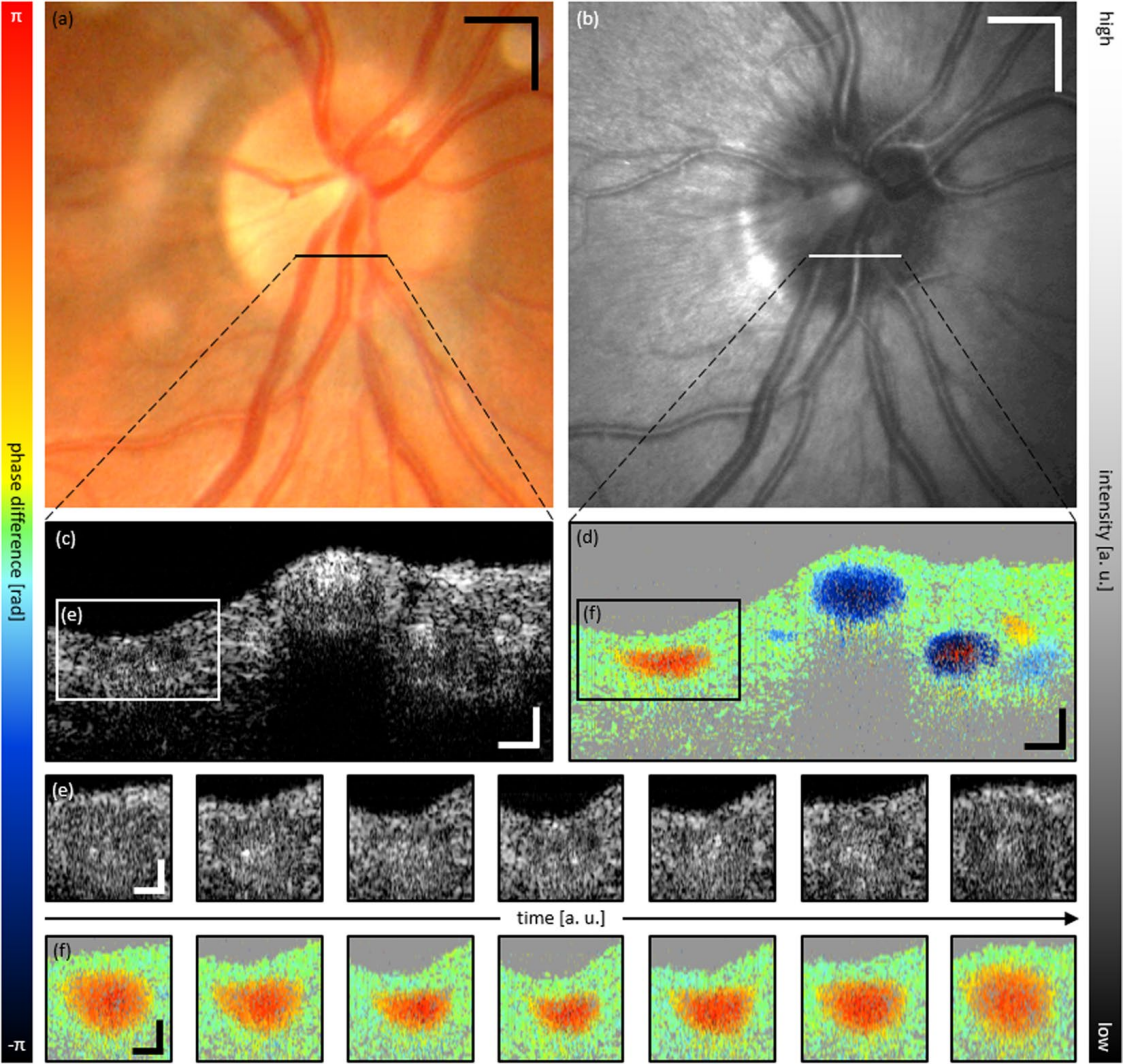
Representative multi-modal image data of SRVP of subject 2. (**a**) Fundus photography and (**b**) SLO image with indicated region of collapse. Cross-sectional OCT (**c**) intensity and (**d**) Doppler tomogram at indicated region of collapse. (**e**,**f**) respective time sequence over full cardiac cycle of ROI indicated in (**c**,**d**). Supplementary Video S4 provides a movie-file of the snapshot images of (**c**,**d**). Scale bars: (**a**,**b**): 0.5 mm; (**c**–**f**): 0.1 mm.

Figure 4 is arranged similarly to Fig. 2 and depicts image data of a partial collapse in an inferior hemivein of a second eye. Supplementary Video S4 presents the corresponding movie (intensity tomograms – top; Doppler tomograms – bottom; 24 fps).

**Figure 5:**
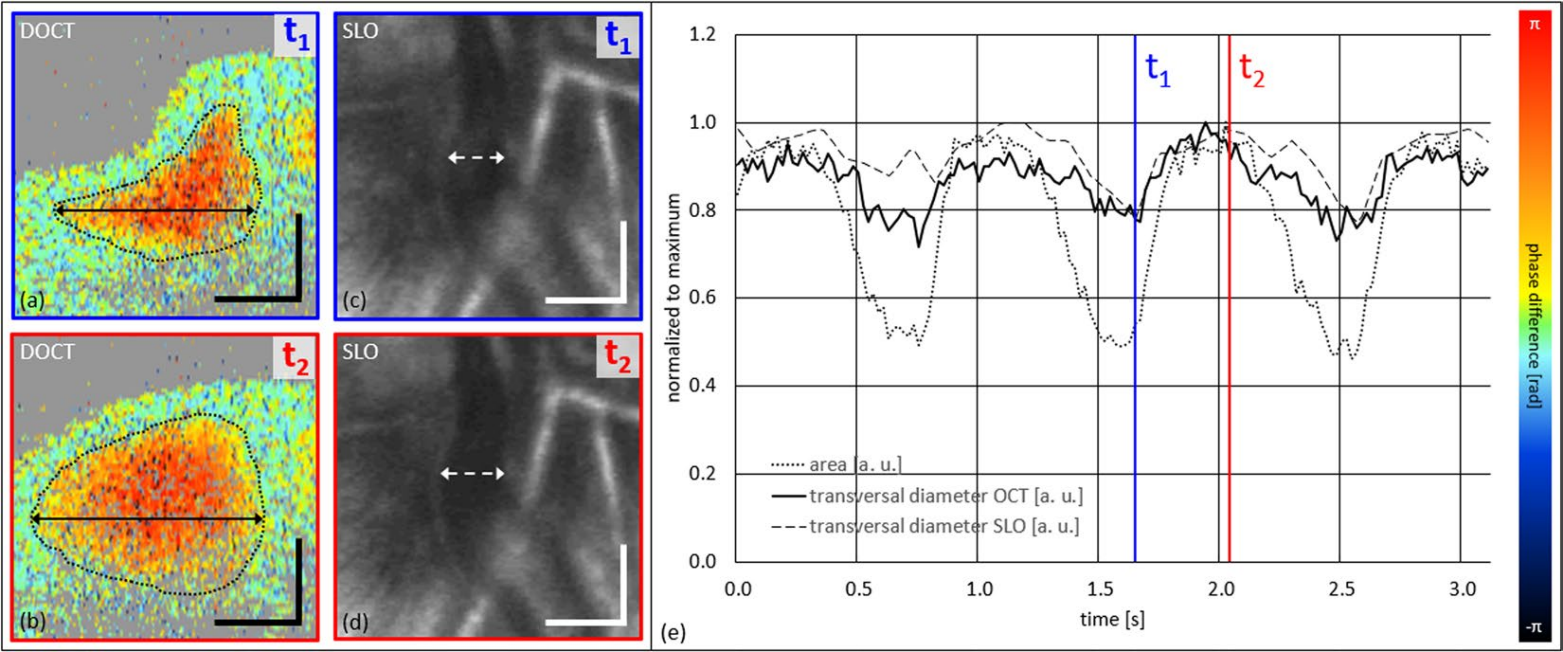
Quantitative SRVP evaluation of DOCT vs. SLO image data over three full cardiac cycles of subject 1 by the expert reader. (**a**) and (**b**) DOCT tomograms at time points of t_1_ and t_2_. (**c**,**d**) SLO images at time points t_1_ and t_2_. Both data sets, DOCT and SLO, were not acquired within one measurement session but within consecutive ones. (**e**) Graph of normalized change of transversal vessel diameter (DOCT and SLO) and normalized change of perfused cross-sectional vessel area (DOCT) with indicated t_1_ and t_2_. Scale bars: (**a**,**b**): 0.1 mm; (**c**,**d**): 0.2 mm.

To compare SRVP quantification between SLO and DOCT, the image sequences of Supplementary Videos S1 and S2 were manually evaluated by an expert reader regarding changes of image features. For this purpose, data acquired over three consecutive cardiac cycles were analyzed. For both sequences the transversal vessel diameter was determined at the site of highest collapse amplitude via marking the vessel’s laterally outmost areas at the borderline between static and dynamic tissue components at both sides (the DOCT and not the intensity data were used for transversal diameter determination). Additionally, the vessel area was determined by manual segmentation of the DOCT data, again at the borderline between static (green) and dynamic (yellow to orange to red) image components. Figure 5 illustrates the manual evaluation procedure with the help of four representative images (Fig. 5(a–d)) and provides the quantitative results in a graph (Fig. 5(e)). DOCT (Fig. 5(a,b)) and SLO (Fig. 5(c,d)) images at two opposing time points of the cardiac cycle (highest amplitude of collapse – t_1_; full venous expansion – t_2_) are depicted. In Fig. 5(e) the normalized measurement results of area and transversal diameter are plotted over the time of ~3.2 s – also indicating t_1_ and t_2_. While the maximum transversal diameter change seems to be roughly 20% for both imaging modalities, the change of segmented area of flow decreases by roughly 50%. Since not every cardiac cycle is similar to the previous one in duration and strength, also the characteristics of venous collapses may change over time. However, in particular the DOCT data shows rather reproducible diameter and area changes over the inspected three consecutive cardiac cycles. For the SLO data especially the first collapse of the sequence seems not as pronounced as the latter two, and thus deviates from the transversal diameter DOCT curve. In general, the transversal diameter changes measured from DOCT and SLO seem to lie within a similar range.

**Figure 6:**
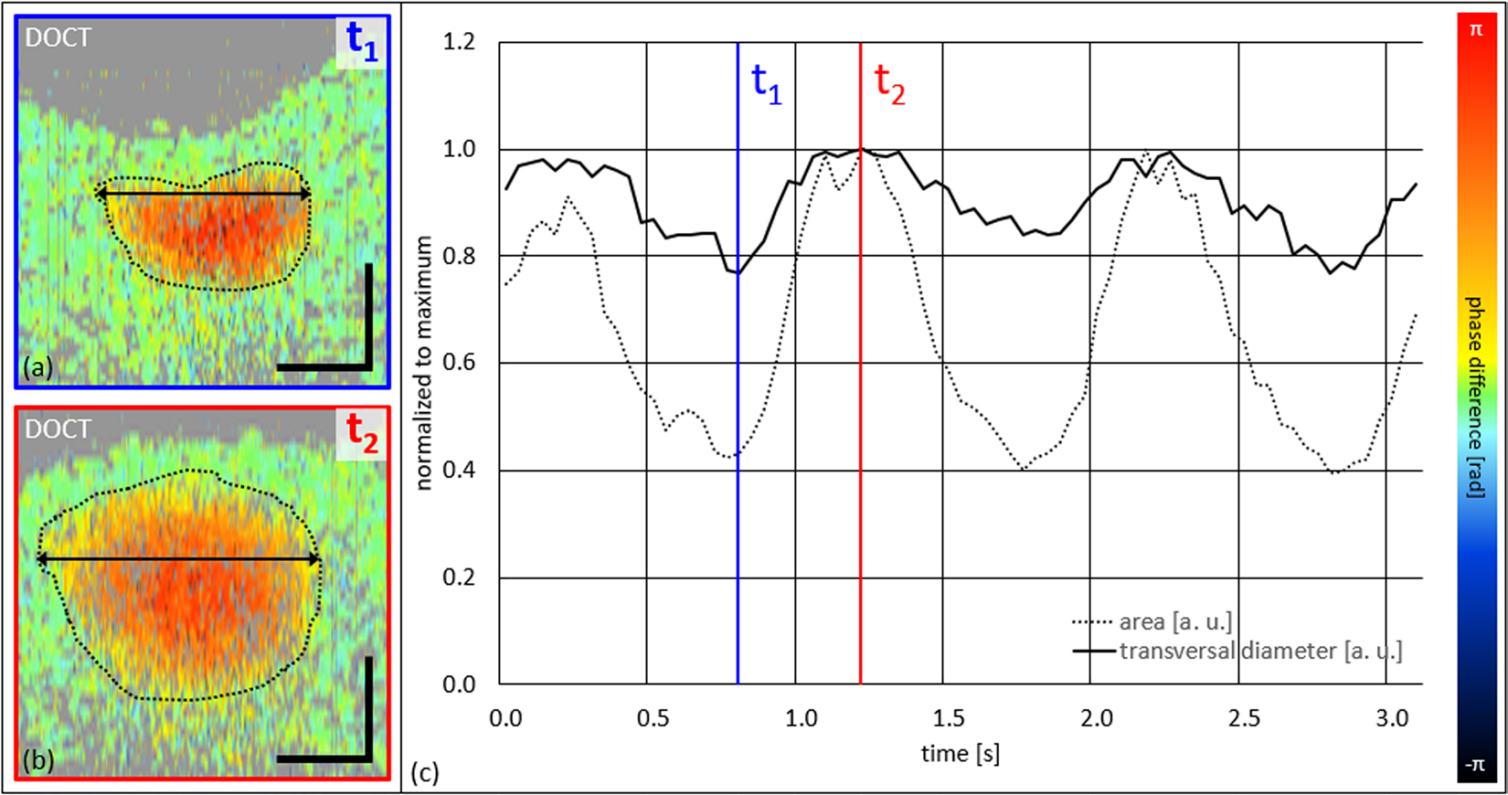
Quantitative SRVP evaluation of DOCT image data over three full cardiac cycles of subject 2 by the expert reader. (**a**,**b**) DOCT tomograms at time points of t_1_ and t_2_. (**c**) Graph of normalized change of transversal vessel diameter and normalized change of perfused cross-sectional vessel area with indicated t_1_ and t_2_. Scale bars: 0.1 mm.

We also quantitatively evaluated Supplementary Video S4 of the second eye showing a partial collapse – cf. Fig. 6. Again, three cardiac cycles were manually evaluated, this time only in terms of the DOCT data. Figure 6(a,b) depict Doppler tomograms at two opposing cardiac time points (highest amplitude of collapse – t_1_; full venous expansion – t_2_). Figure 5(c) plots the normalized measurement results of area and transversal diameter over the time of ~3.2 s – also indicating t_1_ and t_2_. The maximum change of transversal diameter is ~20%, while the maximum change of flow area is ~60% in this case. Thus, the detection sensitivity regarding SRVP is increased by a factor of ~3 by DOCT when comparing relative changes of transversal vessel diameter to relative changes of venous cross-sectional area.

### Venous caliber pulsation vs. venous flow pulsation

**Figure 7:**
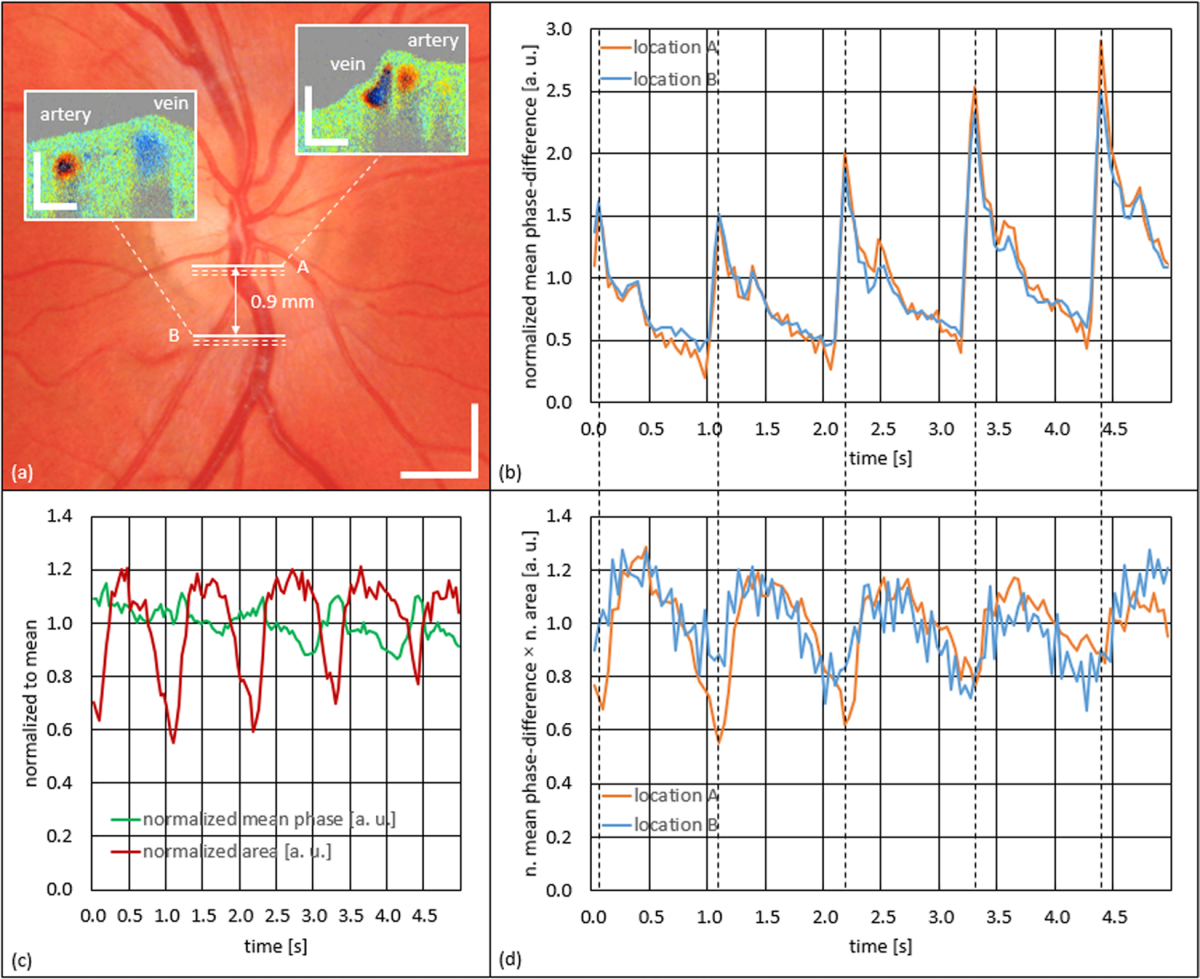
Time-resolved investigation of venous caliber pulsations and venous and arterial flow pulsations using the double-channel imaging mode over five cardiac cycles. (**a**) Fundus photography with indications of measurement locations (A and B) and respective DOCT tomograms. (**b**) Graph of arterial flow pulsation at locations A (orange curve) and B (blue curve) presenting a typical arterial pulse profile. (**c**) Graph of phase-difference change (green curve) and change of cross-sectional vessel area (red curve) of the collapsing vein at location A. (**d**) Graph of venous flow oscillations at location A (orange curve) and B (blue curve). Supplementary Video S5 provides the movie-file of the evaluated data set. Scale bars: 1 mm; insets: 0.25 mm.

Besides caliber pulsations, we also found flow pulsations in the same retinal veins that exhibit SRVP, as illustrated in Fig. 7. Here, the double-channel mode of our instrument (at 50 kHz A-scan rate) was used to simultaneously study the collapsing vein at the site of collapse (A) and at a location inferior (B). Due to individual vasculature arrangement of the imaged eye, the same data set also enabled investigation of an artery at the two referred locations. Figure 7(a) depicts the same fundus photography already presented in Fig. 2(a). The bold white horizontal lines indicate the retinal locations of the two linear B-scans – A and B. The dashed white lines indicate a slight vertical drift towards inferior throughout the 5.0 s acquisition time. Additionally, two representative Doppler tomograms are displayed, where the larger caliber vessel in both images represents the vein whereas the smaller vessel represents the artery which crosses beneath the vein close to location A. Figure 7(b–d) present the quantitative results of the total measurement sequence which is provided in Supplementary Video S5 (24 fps). This movie contains the two simultaneously acquired intensity tomograms (left) at location A (top) and location B (bottom) as well as the corresponding Doppler tomograms (right). Besides the obvious caliber oscillations in the top venous cross-section also changing flow velocity patterns are noticeable in both arterial as well as venous cross-sections.

To obtain a reference regarding the flow evaluation of the collapsing vein, the artery was evaluated first. Figure 7(b) depicts the pulsatile flow changes of the artery over ~5 s for locations A and B, respectively. As expected for an artery, a very distinct pulse-shaped pattern can be observed. The increase in amplitude in both curves towards the end of the measurement is attributed to a change of the Doppler angle due to the mentioned inferior drift of the B-scan position over time. No significant phase delay between the two pulsatile curves could be determined. Thus, the arterial pulse wave velocity (PWV) seems too high to allow quantification at the given measurement parameters of 24 fps and ~0.9 mm distance between measurement locations.

In a second step, venous flow pulsations were evaluated. In comparison to the arterial pulsations, they were considerably weaker in amplitude and showed a more washed-out pulse profile. Figure 7(c) depicts the quantitative results of the collapsing venous segment at location A. The red curve tracks the cross-sectional area changes over time (as previously in Figs 5 and 6), while the green curve tracks changes of flow velocity. Interestingly, an opposing pulsatile behavior was observed among the two curves. As the measurement location is slowly drifting inferiorly, the mean flow velocity change seems to increase as the amplitude of caliber change decreases. The maximum mean flow velocity seems to be in phase with the collapse.

Figure 7(d) plots the normalized mean phase-difference × the normalized area (this product is proportional to the flow through the respective cross-section) of the collapsing vein at locations A and B. At location B no cross-sectional area change is observed, thus, the blue curve is equivalent to the change in velocity. However, at location A strong caliber changes are observed. Hence, the orange curve combines the two curves of Fig. 7(c) to incorporate the changing caliber for flow determination. The blue and orange curve both show flow oscillations ranging within ±20%, and also similar pulsatile profiles which suggests that conservation of blood flow is not violated at the site of collapse. Furthermore, by comparing Fig. 7(b) and (d) one can observe that the peak of the arterial pulse wave coincides with the time point of highest collapse amplitude in the vein (cf. dashed black lines). Similar to the artery, the PWV of the considerably weaker venous flow pulsations could not be determined.

### Pulsatile intensity changes

**Figure 8:**
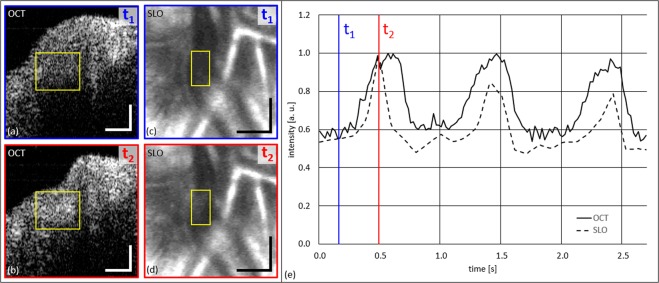
Investigation of pulsatile intensity changes at site of SRVP over three full cardiac cycles. (**a**,**b**) OCT tomograms at time points of t_1_ and t_2_. (**c**,**d**) SLO images at time points t_1_ and t_2_. (**e**) Graph of normalized (to maximum) change of intensity (OCT and SLO) within the yellow ROIs. Scale bars: (**a**,**b**): 0.1 mm; (**c**,**d**): 0.2 mm.

In case of venous collapse, we observed pulsatile intensity changes within the venous lumen. This effect is notable in Figs 2, 4 and Supplementary Videos S1, S4 and S5 and results in intensity changes within a cardiac cycle, synchronous to the vessel collapse. At the time point of highest amplitude of collapse hyperreflectivity is observed within specific areas of the venous cross-section. To illustrate this effect, we tracked the intensity changes in a specific ROI in the intensity tomogram of an additional scan recoded in the same eye at the same retinal location as presented in Fig. 2. Figure 8 presents the mean intensity change within the indicated yellow ROI over three cardiac cycles. Figure 8(a,b) depict snapshot images of two opposing cardiac time points (full venous expansion – t_1_; highest amplitude of collapse – t_2_) and Fig. 8(e) plots the normalized mean intensity change over the time of ~2.7 s. Figure 8 also analyses the changes in SLO reflectivity of the same venous collapse over roughly three cardiac cycles. Again, Fig. 8(c,d) depict snapshot images of the two time points, while the quantitative evaluation of the normalized mean intensity within the indicated yellow ROIs is presented in Fig. 8(e). Supplementary Video S6 presents the three cardiac cycles long sequence of SLO and OCT data used for this analysis. The similar pulsatile behavior suggests that both intensity oscillations – in OCT and SLO data – arise from the same phenomenon.

## Discussion

Within this pilot study, we present first time-resolved quantitative OCT investigations of the phenomenon of SRVP in healthy subjects, using both the single- and double-channel imaging mode of our multi-channel instrument. The tomographic imaging approach and, in particular, the functional contrast enabled by DOCT, facilitate detailed investigations of the perfusion characteristics over time, unmatched by previous reports of SRVP. (i) The quantitative measurement of the SRVP amplitude was improved by a factor of ~3 when relative changes of transversal vessel diameter are compared to relative changes of venous cross-sectional area. Thus, the previous hypothesis of a much stronger caliber change in axial than in lateral direction could be confirmed^21–23^. (ii) The same venous caliber oscillations were imaged repeatedly in several imaging sessions on different days, tracked over multiple cardiac cycles and the detected venous flow pulsations were analyzed. (iii) Pulsatile changes of intensity, probably due to alterations of red blood cell (RBC) orientation, were detected, quantitatively evaluated, and compared to reports of modified photo-plethysmography/densitometry.

The major limitation of this study is its small sample size. We present image data of only two partial retinal venous collapses in two healthy subjects. However, in the course of the study a total of twelve eyes of six healthy subjects (two female, four male; age range: 24–60 yrs) were imaged. Nevertheless, we did not find signs of partial or total venous collapse in any of the veins of the other ten eyes (including the contralateral eyes of the two SRVP exhibiting eyes). We did not include imaging results of those eyes, since time-resolved DOCT investigations of non-collapsing veins have been reported multiple times^31,33,35–42^, and would add little additional value to this study. This observation stands in clear contradiction to previously reported occurrence rates of SRVP in up to 98% of the investigated eyes^3–6^. However, as pointed out in this work, these high occurrence rates were determined by use of enface imaging modalities such as fundus photography/videography and SLO. Slight intensity changes associated with fundus pulsations in the ONH area might be mistaken for SRVPs and potentially lead to overestimation, if the depth information is not available. We also observed such small intensity changes throughout the entire ONH area for all SLO recordings of the ten non-SRVP eyes, however those originated from fundus pulsations^43^ and not from SRVP. Future evaluations are needed to study SRVP using DOCT in a larger sample size to extend the findings presented in this pilot study. However, the lack of commercial DOCT instruments presently impedes clinical applicability for larger scale studies. In this regard SLO is a step ahead of DOCT, while also being the less complex approach. Nevertheless, given the recent success of OCT angiography that has already been implemented in several commercially available instruments, a similar development for quantitative DOCT in the near future does not seem unrealistic.

A potential systematic error of the presented investigations may arise from the manual evaluation of cross-sectional flow area and transversal diameter of the vein. However, all reported area and diameter data were evaluated by the same expert reader. Therefore, such systematic segmentation errors would only have little impact on the results of relative changes, since it can be assumed that the reader’s systematic deviation would be similar for all phases of the cardiac cycle. Furthermore, manual retinal vessel segmentation and diameter evaluation in OCT data by an expert reader is state-of-the-art for ground truth determination and also reported to achieve accurate results in comparison to automated computational segmentation algorithms^44,45^.

In the literature SRVP is termed a ‘pulsation’ due to the fact that pulsatile changes in venous vessel caliber were observed^1,2^. However, in cardiovascular research the term ‘pulsation’ is not necessarily restricted to caliber oscillations but equivalently used for rhythmical changes of blood volume throughput or blood flow in non-collapsing vessels. Such flow pulsations are commonly observed throughout the arterial cardiovascular system and well described in the literature – also in the retina^36,37,46^. Venous flow pulsations are in general not observable^47–50^, and if occurring, restricted to specific locations within the human body. Venous flow pulsations have been reported in the internal jugular vein^51^, the brain^52^ and the retina^36–39^, although, reports are controversial in the latter case^40–42,46^. In contradiction to some of those studies, but in conformity to others, we did measure significant flow pulsations in retinal veins. However, we did not succeed in determination of the travel direction of the observed pulsation. Thus, the hypothesis of upstream pulse propagation of a wave that is generated in the optic nerve subarachnoid space and travels opposite to the direction of blood flow through the lamina cribrosa towards the retina, could neither be verified nor falsified^1,49,53^. The temporal resolution in our experiments was too low (or the maximum channel separation distance was too short) to reasonably evaluate the recorded pulse profiles.

We were also unable to quantify arterial PWV – a recently critically discussed retinal perfusion parameter^39,54–57^ – using imaging parameters of 24 fps and ~0.9 mm distance between the measurements locations. This is in so far interesting, as a recently published article reports to do so, using similar measurement parameters but a different scanning method, called jump-scanning^56^. However, in that report the authors miss to explain how heart rate variability – the physiological variation in the time interval between two consecutive heartbeats – is taken into account. The results of their PWV measurements would be affected severely if this variation was not compensated for^57^. We believe, our technique of simultaneous data recording at separate vessel locations using multi-channel OCT, offers great potential to narrow the high range of previously published arterial PWV values down considerably^39,54–56^. In the future this could be enabled either by an increase of B-scan frame-rate or a larger separation distance between respective measurement locations.

Modified photo-plethysmography/densitometry is described to assess variations in the axial blood column over time by monitoring changes of absorption during fundus videography^1,24,25^. This technology measures the fluctuating light attenuation by blood during a cardiac cycle. The changes in attenuation are then directly related to changes of vessel cross-sections during SRVP using the Beer-Lambert law, assuming that the attenuation is caused by absorption of light by the hemoglobin of the RBCs. However, this assumption ignores a fundamental physical process that needs to be considered for such investigations – scattering. Modified photo-plethysmography/densitometry has no means to differentiate whether the observed pulsatile changes in backreflected intensity originate from changes in absorption or from orientation changes of the scattering particles – in this case RBCs. However, OCT enables such differentiation by measuring depth resolved scattering profiles. The scattering characteristics of moving RBCs that is of importance here, has been described in detail in the literature^58–62^. RBCs are reported to show preferential orientations for laminar flow inside a tubular lumen, as long as a sufficiently high flow velocity is maintained. This particular spatial arrangement results in a so-called hourglass signal pattern in the OCT intensity tomogram of a vessel cross-section. However, this signal pattern was only reported for laminar flow in static tubular organs. In Fig. 7 we analyze what we believe to be pulsatile disturbances of this preferential alignment of RBCs. Additionally, we provide en-face SLO data that is very much comparable to data recorded during modified photo-plethysmography/densitometry (admittedly, there is a difference in the respective wavelength regime). We believe the similar intensity oscillations observed in the imaging data of both modalities to arise from the same phenomenon, and thus conclude that most of the observed pulsatile signal change is due to changes in scattering of RBCs and not absorption. Thus, the assumption of Morgan *et al*.^1^ that variation in hemoglobin is the only pulsatile component is incomplete. The reason for the pulsatile intensity changes is not just the change in axial blood column. The change in orientation of scattering RBCs seems to have a larger effect and must be taken into account. Hence, the quantitative results obtained by modified photo-plethysmography/densitometry are questionable – this technique does not allow for reliable measurements of changing optical path length by consideration of absorption only.

A method to non-invasively assess ICP would denote a milestone for monitoring and management of numerous neurological disorder and conditions^7,15,16^. The introduction of time-resolved DOCT for investigation of SRVP might assist the proposed optical methods for potential future non-invasive ICP assessment. That being said, certain doubts about whether the phenomenon of SRVP might eventually enable reliable ICP determination have to be expressed. We experienced a very strong inter-subject variability in terms of location, strength and characteristics of SRVP (and also minor inter-pulse variability at the site of collapse). Such strong variability may prohibit direct use of SRVP for ICP determination. However, the induction of non-spontaneous artificial retinal vein pulsations by means of ophthalmodynamometry^2,4,9–11^ may solve this problem. Besides potential ICP determination, monitoring of SRVP might proof beneficial for analysis of major ocular disorders such as glaucoma or venous occlusions^1,8^ that have been associated with vascular pathologies.

## Conclusion

In this manuscript, we present first time-resolved investigations of SRVP using intensity-based OCT as well as functional DOCT. Both, the single- and the double-channel imaging mode of our multi-channel OCT prototype enable detailed qualitative as well as quantitative analysis of venous caliber oscillations. The presented OCT image data is compared to standard SLO en-face image data emphasizing the advantages of a tomographic imaging principle for SRVP investigations. Besides pure caliber oscillations also venous flow pulsatility is analyzed in the eyes of healthy volunteers and previously established theoretical hypotheses examined. We hope this first demonstration in combination with potential more elaborate future studies might assist to better understand the role of SRVP for non-invasive ICP assessment as well as for diagnosis and monitoring of certain retinal pathologies.

## Supplementary information

Supplementary Video S1

Supplementary Video S2

Supplementary Video S3

Supplementary Video S4

Supplementary Video S5

Supplementary Video S6

## Data Availability

The data sets generated and analyzed during the current study are available from the corresponding author on reasonable request.

## References

[1] Morgan WH, Hazelton ML, Yu D-Y (2016). Retinal venous pulsation: Expanding our understanding and use of this enigmatic phenomenon. Prog. Retin. Eye Res..

[2] Jacks AS, Miller NR (2003). Spontaneous retinal venous pulsation: aetiology and significance. J. Neurol. Neurosurg. Psychiatry.

[3] Lorentzen SE (1970). Incidence of spontaneous venous pulsation in the retina. Acta Ohthalmol..

[4] Levin BE (1978). The clinical significance of spontaneous pulsations of the retinal vein. Arch. Neurol..

[5] Morgan WH (2004). Retinal venous pulsation in glaucoma and glaucoma suspects. Ophthalmology.

[6] Harder B, Jonas JB (2007). Frequency of spontaneous pulsations of the central retinal vein. Br. J. Ophthalmol..

[7] Jonas JB, Wang N, Yang D, Ritch R, Panda-Jonas S (2015). Facts and myths of cerebrospinal fluid pressure for the physiology of the eye. Prog. Retin. Eye Res..

[8] Golzan SM, Avolio A, Graham SL (2012). Hemodynamic interactions in the eye: a review. Ophthalmologica.

[9] Meyer-Schickerath R, Kleinwaechter T, Papenfuss HD, Firsching R (1995). Central retinal venous outflow pressure. Graefes Arch. Clin. Exp. Ophthalmol..

[10] Firsching R, Schütze M, Motschmann M, Behrens-Baumann W (2000). Venous ophthalmodynamometry: a noninvasive method for assessment of intracranial pressure. J. Neurosurg..

[11] Firsching R (2011). Noninvasive assessment of intracranial pressure with venous ophthalmodynamometry. J. Neurosurg..

[12] Golzan SM, Kim MO, Seddighi AS, Avolio A, Graham SL (2012). Non-invasive estimation of cerebrospinal fluid pressure waveforms by means of retinal venous pulsatility and central aortic blood pressure. Ann. Biomed. Eng..

[13] Morgan WH (2012). Retinal vein pulsation is in phase with intracranial pressure and not intraocular pressure. Invest. Ophthalmol. Vis. Sci..

[14] Wong SH, White RP (2013). The clinical validity of the spontaneous retinal venous pulsation. J. Neuroophthalmol..

[15] Zhang X (2017). Invasive and noninvasive means of measuring intracranial pressure: a review. Physiol. Meas..

[16] Bruce BB (2014). Noninvasive assessment of cerebrospinal fluid pressure. J. Neuroophthalmol..

[17] Lam J (2016). Structural characteristics of the optic nerve head influencing human retinal venous pulsations. Exp. Eye Res..

[18] Golzan SM, Graham SL, Leaney J, Avolio A (2011). Dynamic association between intraocular pressure and spontaneous pulsations of retinal veins. Curr. Eye Res..

[19] Moret F, Poloschek CM, Lagrèze WA, Bach M (2011). Visualization of fundus vessel pulsation using principal component analysis. Invest. Ophthalmol. Vis. Sci..

[20] Seo JH (2012). Relationship of intraocular pressure and frequency of spontaneous retinal venous pulsation in primary open-angle glaucoma. Ophthalmology.

[21] Moreno AH, Katz AI, Gold LD, Reddy RV (1970). Mechanics of distension of dog veins and other very thin-walled tubular structures. Circ. Res..

[22] Heil M (1997). Stokes flow in collapsible tubes: computation and experiment. J. Fluid Mech..

[23] Moret F, Reiff CM, Lagreze WA, Bach M (2015). Quantitative analysis of fundus-image sequences reveals phase of spontaneous venous pulsations. Transl. Vis. Sci. Technol..

[24] Morgan WH (2014). Photoplethysmographic measurement of various retinal vascular pulsation parameters and measurement of the venous phase delay. Invest. Ophthalmol. Vis. Sci..

[25] Morgan WH (2015). Objective detection of retinal vessel pulsation. PLoS One.

[26] Fercher AF, Drexler W, Hitzenberger CK, Lasser T (2003). Optical coherence tomography - principles and applications. Rep. Prog. Phys..

[27] Drexler W, Fujimoto JG (2008). State-of-the-art retinal optical coherence tomography. Prog. Retin. Eye Res..

[28] Golzan SM, Morgan WH, Georgevsky D, Graham SL (2015). Correlation of retinal nerve fibre layer thickness and spontaneous retinal venous pulsations in glaucoma and normal controls. PLoS One.

[29] Leitgeb RA, Werkmeister RM, Blatter C, Schmetterer L (2014). Doppler optical coherence tomography. Prog. Retin. Eye Res..

[30] Wartak A (2017). Sequential multi-channel OCT in the retina using high-speed fiber optical switches. Proc. SPIE.

[31] Wartak A, Beer F, Baumann B, Pircher M, Hitzenberger CK (2018). Adaptable switching schemes for time-encoded multichannel optical coherence tomography. J. Biomed. Opt..

[32] Trasischker W (2013). *In vitro* and *in vivo* three-dimensional velocity vector measurement by three-beam spectral-domain Doppler optical coherence tomography. J. Biomed. Opt..

[33] Wartak A (2016). Active-passive path-length encoded (APPLE) Doppler OCT. Biomed. Opt. Express.

[34] Suehira N (2012). Three-beam spectral-domain optical coherence tomography for retinal imaging. J. Biomed. Opt..

[35] Haindl R (2016). Total retinal blood flow measurement by three beam Doppler optical coherence tomography. Biomed. Opt. Express.

[36] Yazdanfar S, Rollins AM, Izatt JA (2003). *In vivo* imaging of human retinal flow dynamics by color doppler optical coherence tomography. Arch. Ophthalmol..

[37] Schmoll T, Leitgeb RA (2013). Heart-beat-phase-coherent Doppler optical coherence tomography for measuring pulsatile ocular blood flow. J. Biophotonics.

[38] Wang Y, Bower BA, Izatt JA, Tan O, Huang D (2007). *In vivo* total retinal blood flow measurement by Fourier domain Doppler optical coherence tomography. J. Biomed. Opt..

[39] Spahr H (2015). Imaging pulse wave propagation in human retinal vessels using full-field swept-source optical coherence tomography. Opt. Lett..

[40] Blatter C (2013). Dove prism based rotating dual beam bidirectional Doppler OCT. Biomed. Opt. Express.

[41] Doblhoff-Dier V (2014). Measurement of the total retinal blood flow using dual beam Fourier-domain Doppler optical coherence tomography with orthogonal detection planes. Biomed. Opt. Express.

[42] Tan O (2015). En face Doppler total retinal blood flow measurement with 70 kHz spectral optical coherence tomography. J. Biomedical Opt..

[43] Augustin M (2017). Ocular fundus pulsations within the posterior rat eye: Chorioscleral motion and response to elevated intraocular pressure. Sci. Rep..

[44] Pilch M (2012). Automated segmentation of retinal blood vessels in spectral domain optical coherence tomography scans. Biomed. Opt. Express.

[45] Ouyang Y, Shao Q, Scharf D, Joussen AM, Heussen FM (2015). Retinal vessel diameter measurements by spectral domain optical coherence tomography. Graefes Arch. Clin. Exp. Ophthalmol..

[46] Riva CE, Grunwald JE, Sinclair SH, Petrig BL (1985). Blood velocity and volumetric flow rate in human retinal vessels. Invest. Ophthalmol. Vis. Sci..

[47] Caro, C. G., Pedley, T. J., Schroter, R. C. & Seed, W. A. The Mechanics of the Circulation. *Cambridge University Press* (2012).

[48] Zweifach BW (1974). Quantitative studies of microcirculatory structure and function. I. Analysis of pressure distribution in the terminal vascular bed in cat mesentery. Circ. Res..

[49] Levine DN (1998). Spontaneous pulsation of the retinal veins. Microvasc. Res..

[50] Gugleta K (2006). On pulse-wave propagation in the ocular circulation. Invest. Ophthalmol. Vis. Sci..

[51] Drazner MH, Rame JE, Stevenson LW, Dries DL (2001). Prognostic importance of elevated jugular venous pressure and a third heart sound in patients with heart failure. N. Engl. J. Med..

[52] Wagshul ME, Eide PK, Madsen JR (2011). The pulsating brain: A review of experimental and clinical studies of intracranial pulsatility. Fluids Barriers CNS.

[53] Levine DN, Bebie H (2016). Phase and amplitude of spontaneous retinal vein pulsations: An extended constant inflow and variable outflow model. Microvasc. Res..

[54] Kotliar KE, Baumann M, Vilser W, Lanzl IM (2011). Pulse wave velocity in retinal arteries of healthy volunteers. Br. J. Ophthalmol..

[55] Kotliar KE (2013). Retinal pulse wave velocity in young male normotensive and mildly hypertensive subjects. Microcirculation.

[56] Li Q (2018). Retinal pulse wave velocity measurement using spectral-domain optical coherence tomography. J. Biophotonics.

[57] Spahr H, Hillmann D, Pfäffle C, Hüttmann G (2018). Comment on “Retinal pulse wave velocity measurement using spectral-domain optical coherence tomography”. J. Biophotonics.

[58] Szkulmowska A, Szkulmowski M, Kowalczyk A, Wojtkowski M (2008). Phase-resolved Doppler optical coherence tomography – limitations and improvements. Opt. Lett..

[59] Srinivasan VJ (2011). Optical coherence tomography for the quantitative study of cerebrovascular physiology. J. Cereb. Blood Flow Metab..

[60] Cimalla P, Walther J, Mittasch M, Koch E (2011). Shear flow-induced optical inhomogeneity of blood assessed *in vivo* and *in vitro* by spectral domain optical coherence tomography in the 1.3 mum wavelength range. J. Biomed. Opt..

[61] Seidel G (2016). Estimating retinal blood flow velocities by optical coherence tomography. JAMA Ophthalmol..

[62] Bernucci MT, Merkle CW, Srinivasan VJ (2018). Investigation of artifacts in retinal and choroidal OCT angiography with a contrast agent. Biomed. Opt. Express.

